# *In vivo* detection of teriflunomide-derived fluorine signal during neuroinflammation using fluorine MR spectroscopy

**DOI:** 10.7150/thno.47130

**Published:** 2021-01-01

**Authors:** Christian Prinz, Ludger Starke, Jason M. Millward, Ariane Fillmer, Paula Ramos Delgado, Helmar Waiczies, Andreas Pohlmann, Michael Rothe, Marc Nazaré, Friedemann Paul, Thoralf Niendorf, Sonia Waiczies

**Affiliations:** 1 Berlin Ultrahigh Field Facility (B.U.F.F.), Max Delbrück Center for Molecular Medicine, Berlin, Germany.; 2 Experimental and Clinical Research Center, a joint cooperation between the Charité - Universitätsmedizin Berlin and the Max Delbrück Center for Molecular Medicine in the Helmholtz Association, Berlin, Germany.; 3 Physikalisch-Technische Bundesanstalt (PTB), Berlin, Germany.; 4 MRI TOOLS GmbH, Berlin, Germany.; 5 Lipidomix GmbH, Berlin, Germany.; 6 Medicinal Chemistry, Leibniz-Institut fϋr Molekulare Pharmakologie (FMP), Berlin, Germany.; 7 Charité - Universitätsmedizin Berlin, corporate member of Freie Universität Berlin, Humboldt-Universität zu Berlin, and Berlin Institute of Health (BIH), Berlin, Germany.

**Keywords:** MRI, MRS, Fluorine, Teriflunomide, Magnetic Resonance Spectroscopy, experimental autoimmune encephalomyelitis, Multiple Sclerosis

## Abstract

**Background:** Magnetic resonance imaging (MRI) is indispensable for diagnosing neurological conditions such as multiple sclerosis (MS). MRI also supports decisions regarding the choice of disease-modifying drugs (DMDs). Determining *in vivo* tissue concentrations of DMDs has the potential to become an essential clinical tool for therapeutic drug monitoring (TDM). The aim here was to examine the feasibility of fluorine-19 (^19^F) MR methods to detect the fluorinated DMD teriflunomide (TF) during normal and pathological conditions.

**Methods:** We used ^19^F MR spectroscopy to detect TF in the experimental autoimmune encephalomyelitis (EAE) mouse model of multiple sclerosis (MS) *in vivo*. Prior to the *in vivo* investigations we characterized the MR properties of TF *in vitro*. We studied the impact of pH and protein binding as well as MR contrast agents.

**Results:** We could detect TF *in vivo* and could follow the ^19^F MR signal over different time points of disease. We quantified TF concentrations in different tissues using HPLC/MS and showed a significant correlation between *ex vivo* TF levels in serum and the *ex vivo* ^19^F MR signal.

**Conclusion:** This study demonstrates the feasibility of ^19^F MR methods to detect TF during neuroinflammation *in vivo*. It also highlights the need for further technological developments in this field. The ultimate goal is to add ^19^F MR protocols to conventional ^1^H MRI protocols in clinical practice to guide therapy decisions.

## Introduction

Multiple sclerosis (MS) is a chronic inflammatory disease of the central nervous system (CNS) 1, 2. The disease course is highly variable, involving a wide spectrum of neurological and motoric symptoms 3. Most patients show a relapsing and remitting (RRMS) disease course, which ultimately transitions to a progressive phase 2, 4.

^1^H Magnetic resonance imaging (^1^H MRI) has been indispensable for diagnosing MS 5-7. ^1^H MRI can distinguish chronic from active lesions when using contrast agents to reveal blood brain barrier (BBB) disruptions 8-11. Furthermore, MRI has been vital for making safe informed decisions with respect to disease modifying drugs (DMDs) 12, 13 to ensure a better dampening of disease activity 14. Typically, T_2_ lesion load 15, 16 and brain atrophy 16 are used as outcome measures, especially during clinical studies. These MRI endpoints are commonly used as primary and secondary measures in phase II DMD trials involving large amounts of patients 17. Despite the substantial armamentarium of DMDs available for MS 18-20, predicting treatment outcomes and tailoring DMD dosages to treatment objectives for individual patients poses a major unmet clinical need 21-23. DMDs may need to traverse the BBB, to modify inflammatory responses within the CNS or to reduce neurodegeneration 24.

Currently there are no standard clinical methods to non-invasively monitor the distribution of drugs in patients. However, the possibility to quantify the concentration of drugs in the brain would greatly improve the assessment of individual treatment responses 22. Drug levels are typically measured in blood, urine, saliva, and infrequently cerebrospinal fluid (CSF). However, drug levels in these fluids do not reliably reflect concentrations within the CNS. Imaging techniques such as positron emission tomography (PET) and single-photon emission computed tomography (SPECT) are used to detect drugs labeled with a radioisotope; these imaging methods are highly sensitive and are particularly useful in phase I clinical studies for small cohorts of human subjects 25-29. However, they are not used for standard drug monitoring in patients; disadvantages include high costs, the necessity to inject radioactive compounds, restricted observation window due to short radiotracer half-life (^18^F t_1/2_ = 110 min), and a lack of distinction between drugs and their metabolites.

One third of all approved drugs are fluorinated, and are potentially detectable by fluorine-19 (^19^F) MRI *in vivo* 30-32. The amount of ^19^F atoms endogenously present in the human body that can be detected with MR methods is negligible. The absence of background signal makes the ^19^F nucleus a unique and highly attractive biomarker for detecting administered fluorinated DMDs *in vivo* using ^19^F-MR methods. ^19^F MR methods have been applied to detect fluorinated drugs in animal 33-38 and human studies 39-45 in the past. They have also been applied in combination with drugs encapsulated in fluorine rich nanoparticles to monitor the efficacy of these therapies in animal models 46. ^19^F-MR methods have also been employed in diagnostic imaging 47, for guiding tumor ablation therapies 48, and for imaging intracellular therapeutic targeting 49. However, they remain under-utilized for the majority of drugs, especially in MS.

Teriflunomide (TF) is an anti-inflammatory DMD approved for use in MS that contains a trifluoromethyl group 50. TF is administered orally once-daily and has a rapid, complete absorption with a long half-life (> 2 weeks) due to extensive enterohepatic recycling 51. In RRMS patients, it reduces the annual relapse rate, slows disability progression, reduces the lesion volume 51, 52 and brain volume loss 53. TF has a high tolerability and low discontinuation rate 50, 54. TF was investigated in preclinical studies using the animal model of MS, experimental autoimmune encephalomyelitis (EAE) 55-57. In rats, TF administration at EAE onset reduced disease severity and delayed progression 55, 57. TF treatment delayed EAE onset in SJL/J mice, and suppressed disease entirely in C57BL/6 mice.

In this study we used ^19^F MR spectroscopy to study the possibility of detecting TF during EAE *in vivo*. We characterized the MR properties of TF *in vitro*, studying the impact of pH, protein binding, and MR contrast agents. We demonstrated the feasibility of ^19^F MR methods to detect TF non-invasively during neuroinflammation, with the ultimate goal for further development into future clinical applications.

## Methods

### Teriflunomide

Teriflunomide (TF) (Sanofi-Genzyme, Bridgewater, US) was formulated in 0.6% carboxymethylcellulose, sodium salt (CMC, Sigma, Schnelldorf, Germany) in the form of a suspension for *in vivo* use. CMC is an inactive ingredient used as thickening excipient, stabilizer and suspending agent. TF (20 mg) was mixed with 5 mL CMC/Tween-80 (0.6% CMC, 0.5% Tween-80 in water) using medium speed magnetic stirring (circa 24 h at RT) until a uniform milky suspension was obtained. The suspension was transferred to a clean glass vial and the original vial rinsed with a further 5 mL of CMC/Tween-80. The TF suspension (2 mg/mL) was adjusted to a pH of 7 using HCl and NaOH. For phantom experiments, TF was prepared in CMC, DMSO and human serum to study the effects of protein binding.

### Animals

Dark Agouti rats (n = 2, Janvier Labs, Le Genest-Saint-Isle, France), C57BL/6N mice (n = 27, Charles River, Sulzfeld, Germany) and SJL/J (n = 33, Janvier Labs, Le Genest-Saint-Isle, France) mice (all female, age 2-4 months) were used to study the TF signal following oral application *in vivo*. The numbers of animals within each group are specified in the relevant experiments below.

Animal experiments were conducted in accordance with procedures approved by the Animal Welfare Department of the State Office of Health and Social Affairs Berlin (LAGeSo) and conformed to guidelines to minimize discomfort to animals (86/609/EEC).

### EAE induction

EAE was induced by subcutaneous immunization of SJL/J mice with proteolipid protein peptide (PLP_139-151_) and C57BL/6 mice with myelin oligodendrocyte glycoprotein peptide (MOG_35-55_); for both peptides 250 µg peptide (Pepceuticals, Leicester, UK) per animal were emulsified with M. Tuberculosis H37RA (List Biological Laboratories, Campbell, US, 800 µg/animal) in 100 µL Complete Freund's Adjuvant (BD Difco, Heidelberg, Germany). Pertussis Toxin (Biotrend, Cologne, Germany, 1.25 ng/µL in SJL/J and 2 ng/µL in C57BL/6) was administered intraperitoneally in 200 µL PBS on days 0 and 2 58.

EAE scoring was performed daily: righting reflex weakness = 0.5, tail paresis = 0.5, tail paralysis = 1, unilateral hindlimb paresis = 0.5, bilateral hindlimb paralysis = 1, unilateral forelimb paresis = 0.75, bilateral forelimb paralysis = 1.5.

### Teriflunomide treatment and preparation for *in vivo* MR measurements

Rats (n = 2) were treated orally with 10 mg/kg TF and MR measurements were performed directly following administration. Animals were anesthetized by intraperitoneal (ip) injection using ketamine (40 mg/kg, WDT, Garbsen, Germany) and medetomidine (0.5 to 0.75 mg/kg, Henry Schein, Berlin, Germany) maintained by an ip catheter line. TF was administered via a catheter line to the stomach while the animal was in the scanner.

Mice were treated daily for 14 days with 30 mg/kg TF 55-57, 59 or vehicle control (CMC) administered by oral gavage. The increased dose used in mice takes into consideration guidelines on dose conversions in animals and is mostly due to differences in metabolism 56, 60. EAE mice were treated with TF (C57BL/6 n = 12, SJL/J n = 12) or CMC (C57BL/6 n = 6, SJL/J n = 6). Healthy non-immunized C57BL/6 (n = 9) or SJL/J (n = 15) mice served as therapy controls. MR measurements in mice were performed on days 8 and 14 following EAE start, 16-24 h after the last drug administration. For *in vivo* MR measurements, mice were anesthetized by intraperitoneal injection using a mixture of xylazine (5 mg/kg, CP Pharma, Burgdorf, Germany) and ketamine (50 mg/kg, WDT, Garbsen, Germany) maintained by an ip catheter line.

Animals were transferred to a temperature-regulated bed (receiving circulated warm water from a water bath) and supplied with pressurized air (30 %) and O_2_ (70 %). Pulse, respiration and body temperature (Neoptix, OmniLink version 1.15, Omniflex, Neoptix, Québec, Canada) were continuously monitored. The body temperature was kept at 37 °C throughout the experiments.

For studying the BBB disruption in SJL/J EAE mice, gadopentetate dimeglumine (0.2 mmol/kg Gd-DTPA Magnevist, Bayer Pharma, Berlin, Germany) was administered intravenously via the tail vein using an infusion pump (Harvard PHD 2000, Harvard Apparatus, Cambridge, US).

### Phantom construction

For characterizing the ^19^F MR properties (chemical shift, spectral shape and relaxation times) of TF, phantoms were prepared in 2.5 mL syringes (inner diameter, id: 9.7 mm, total length: 7.6 cm, B.Braun, Melsungen, Germany) equipped with stopper closing-cones (B.Braun, Melsungen, Germany) using dimethylsulfoxide (DMSO, Roth, Karlsruhe, Germany, 27.02 mg/mL), human serum (4.84 mg/mL) or CMC (2.70 mg/mL) as solvents/suspending agent. Given the different pH of various compartments *in vivo*, we studied the influence of pH on the relaxation times T_1_ and T_2_ as well as the ^19^F signal intensity in CMC in 1 mL syringes (id: 4.7 mm, total length: 9.6 cm, B.Braun, Melsungen, Germany). The pH for the 2.70 mg/mL TF concentration was adjusted to pH values of 5, 7.4, 10 and 13 with HCl or NaOH.

For studying the influence of contrast agent on ^19^F MR properties, 4 phantoms containing 16.67 mg/mL TF and different concentrations of Gd-DTPA (0.5 mM, 1 mM, 2 mM, and 4 mM) in DMSO were prepared in NMR tubes (id: 4.2 mm).

For assessing the limit of detection (LOD) for ^19^F MRS and performing calibrations, four TF concentrations were prepared in serum (400 µL) and the exact concentration for each sample was determined by mass spectrometry (11.8, 105.7, 787.4, 4208.2 µg/g). The serum samples (350 to 500 µL) were prepared in 1 mL syringes (as above).

### MR methods

#### Hardware

MR experiments were performed on a Bruker Biospec 9.4 T MR scanner (Bruker Biospin, Ettlingen, Germany) with a horizontal bore. A room temperature (RT) dual-tunable ^19^F/^1^H head RF transceive coil (16 mm inner diameter) 61 was used to characterize TF in DMSO/serum/CMC in phantom experiments.

A RT dual-tunable ^19^F/^1^H rat body RF transceive coil (MRI.TOOLS GmbH, Berlin, Germany, 62 mm inner diameter) was used to study *in vivo* ^19^F MR spectroscopy (MRS) signals in the abdomen of the rat.

A cryogenically-cooled transceive ^19^F quadrature RF surface probe (20 mm inner diameter, Cryogenic Radiofrequency Probe, CRP, Bruker, Fällanden, Switzerland) 62 was used for *in vivo* ^19^F MRS measurements of the mouse head and abdomen as well as serum samples. With this coil we had previously shown that ^19^F MR sensitivity is enhanced by a factor of 15 compared to RT head coils 62. The bed of the ^19^F CRP was adjusted with respect to the surface of the coil in order to acquire ^19^F MRS in different regions of the mouse body. The measurement volume above the bed was adjusted with respect to the surface of the coil-head by using a position gauge. This device reproduces the geometry of the coil-head and supporting components and can be used to adjust the position of the mouse on the bed outside of the MR scanner. Anatomical ^1^H scans ensured correct positioning and complete coverage of the regions of interest.

#### Phantom MR measurements

A non-selective single-pulse ^19^F MRS FID-acquire sequence (TR = 1000 ms, nominal flip angle = 90°, blockpulse, 4096 sampling points, acquisition delay = 0.05 ms, excitation pulse bandwidth = 10000 Hz, spectral read bandwidth = 25000 Hz, averages for serum/CMC/DMSO phantoms: avg_serum_ = 8, avg_CMC_ = 16, avg_DMSO_ = 16) was used for detecting TF in phantoms and studying chemical shift and spectral shape (full-width half maximum, FWHM). This sequence (later referred to as default) was slightly modified e.g. using increased averages or bandwidth to increase signal-to-noise ratio (SNR) in *in vivo* and *ex vivo* experiments (see below).

^19^F T_1_ and T_2_ relaxation times were measured using MR spectroscopy. For T_1_, the default FID-acquire sequence was used but with 16 TRs ranging from 100 to 10000 ms, avg_serum_ = 40, avg_DMSO_ = 30, avg_CMC_ = 30. For T_2_, a CPMG pulse sequence was used: 25 echoes, echo spacing for serum/CMC/DMSO phantoms: es_serum_ = 2.8 ms, es_DMSO_ = 40 ms, es_DMSO+Gd-DTPA_ = 10.6 ms, es_CMC_ = 10.6 ms, excitation pulse = 5000 Hz, spectral read bandwidth = 25000 Hz; TR_serum_ = 2000 ms, TR_DMSO_ = 5000 ms, TR_CMC_ = 5000 ms, avg = 50 (for all phantoms).

For studying the influence of pH on the ^19^F MR signal detection, the default FID-acquire sequence was used, but a long TR of 8000 ms was chosen to allow full relaxation.

The LOD for the ^19^F CRP to perform ^19^F MRS *in vivo* was assessed using the above four concentrations of TF in serum and the default ^19^F MRS FID-acquire sequence but using avg = 1024, acquisition time = 17 min. The SNR of these spectra was measured and the LOD was determined as the concentration/number of ^19^F atoms that corresponded to an SNR of 5 (SNR_1_ estimation below) using a linear fit with y-axis intercept = 0. The SNR value of 5 was chosen as a conservative threshold for determining LOD.

#### *In vivo* ^1^H MRI using ^19^F/^1^H coils

Anatomical ^1^H MRI was performed using FLASH (Fast Low-Angle Shot) 63 and T_2_ weighted TurboRARE (Rapid Acquisition with Relaxation Enhancement) 64 pulse sequences. In EAE mice, BBB disruptions were assessed using an MDEFT (Modified Driven-Equilibrium FT) 65 sequence with inversion (TR/TE/TI 2600/3.9/950 ms, FOV (30.2×12.8×9) mm^3^, matrix size = 256×170×18, avg = 2, acquisition time = 3 m 7 s).

#### *In vivo* ^19^F MRS using ^19^F CRP

TF-derived ^19^F MR signal was studied in healthy and EAE mice immediately following acquisition of the anatomical scans. To account for the B_1_ inhomogeneity of the CRP surface coil during *in vivo* measurements, we calibrated the flip angle. Before *in vivo* measurements, a phantom reference sample of 1 mL TF in DMSO (27.02 mg/mL) was used to manually calibrate the flip angle and reference power. This sample was in a 2.5 mL syringe and was positioned at the coil surface. We acquired 10 spectra with the default ^19^F MRS FID-acquire sequence using different reference powers (0.0001 - 0.01 W). The best reference power from these manual measurements was verified by the automatically adjusted power settings for this sample by the MR system. Prior to each *in vivo* measurement, we adjusted the reference power manually (automatic ^19^F adjustments were not possible due to low SNR): spectra (avg = 128, 2 min 8 s each) with different reference powers (0.001, 0.002, 0.004, 0.008 W) were acquired from the head region of mice *in vivo* using the default FID-acquire method. The optimal reference power yielding the highest signal intensity was then chosen for the ^19^F MRS data acquisitions. For these acquisitions, the default FID-acquire sequence was used with avg = 1024, acquisition time = 17 min.

#### *In vivo* ^19^F MRS using ^19^F/^1^H rat body coil

To study ^19^F MRS signal of TF in the abdominal region of rats at different time points, the default FID-acquire sequence was used with alterations in: TR = 1500 ms, avg = 256, acquisition time = 6 min.

### *Ex vivo* measurements

#### Tissue processing

Animals were sacrificed after 14 days of *in vivo* experiments. Under deep anesthesia, blood was withdrawn and animals were transcardially perfused with 30 mL (> 10× the estimated total mouse blood volume) phosphate-buffered saline (PBS) solution. CSF was collected from the cisterna magna and the perfused brain was isolated. The perfused brain samples are mostly depleted of blood and CSF. Samples were frozen (-80 °C) for subsequent mass spectrometry studies.

Perfused brain tissue (50 mg), serum (50 µL) or CSF (1-3 mg) was weighed/measured and homogenized in 450 µL phosphate buffer (100 mmol/L, pH = 6.0). 1 mL ethylacetate was added. The mixture was shaken vigorously for 5 min and centrifuged at 11000 rpm for 10 min. The upper layer was transferred to a 2 mL glass vial. The extraction was repeated twice. The organic extract was evaporated to dryness with a gentle N_2_ stream at 40 °C, after which the residue was dissolved in 1 mL ethanol.

#### High performance liquid chromatography mass spectrometry (HPLC/MS)

For assessing the LOD for HPLC/MS, 5 TF concentrations (1 ng/mL to 1000 ng/mL) were prepared in DMSO. LOD was calculated at an SNR = 9 using peak-to-peak algorithm from lowest calibrator 1 ng/mL.

HPLC-measurements were performed using an Agilent 1290 HPLC system with binary pump, autosampler and column thermostat equipped with a Phenomenex Kinetex-C18 column 2.6 µm, 2.1×150 mm column (Phenomenex, Aschaffenburg, Germany). Ammonium acetate (5 mM) and acetonitrile was used as solvent system. All solvents and buffers in HPLC-MS-grade were obtained from VWR Germany. The solvent gradient started at 5 % acetonitrile and was increased to 95 % within 5 min until 8 min with a flow rate of 0.4 mL/min and 1 µL injection volume. The HPLC was coupled with an Agilent 6470 triplequad mass spectrometer with electrospray ionization source using established parameters (gas temp = 250 °C, gas flow = 9 L/min, nebulizer pressure = 20 psi, sheath gas temp = 390 °C, sheath gas flow = 12 L/min, capillary voltage = 2700 V, nozzel voltage = 300 V) operated in negative multiple reaction monitoring mode (269.2 -160 capillary electrophoresis (CE) 28 V, - 82 CE 21, fragmentor voltage = 120 V, mass resolution wide/wide).

#### *Ex vivo* ^19^F MRS

For correlating the TF-derived ^19^F MR signal with HPLC/MS TF quantification, calibrations were first performed with mouse sera spiked with TF (using 3 concentrations closer to biologically expected values) in 1 mL syringes. These concentrations were measured with HPLC/MS (11.8, 105.7, 787.4 µg/g) and the default ^19^F MRS FID-acquire method (but excitation pulse = 70000 Hz, spectral read bandwidth = 70000 Hz, avg = 4096, acquisition time = 1 h 8 min). Next we measured the *ex vivo* serum samples from EAE mice (n = 10) in 1 mL syringes using the default FID-acquire method and HPLC/MS.

For both the calibration experiment (spiked serum) as well as the between-method correlation (*ex vivo* serum) we computed a linear fit with y-axis intercept = 0 to determine the relation of signal to concentration and then used this ratio to estimate TF concentrations in *ex vivo* serum samples from the ^19^F MRS signal intensity after accounting for slight volume differences.

### MR data analysis

For *in vivo* proton image processing and analysis, the freely available software Fiji (Image J v1.47p, Open source software, NIH, MD, USA) 66 was used.

All spectral analyses and processing were performed in MATLAB (The MathWorks Inc., Natick, USA). Chemical shifts are referenced to trichloro-fluoro-methane, CFCl_3_ (δ_F_=0 ppm). Post-processing of the real spectra included zero-filling to 2^14^ points of all original FID data and a line-broadening of 70 Hz. The signal from both receive channels was averaged after zeroth and first order phase-correction.

We used two conventions to measure SNR of the main spectral peaks: SNR_1_ was measured by calculating the ratio of the peak amplitude (maximal peak height minus mean background signal) and one standard deviation of the background noise (*σ*_1_), as suggested in a recent expert's consensus paper on *in vivo* MR spectroscopy 67. SNR_2_ was measured by calculating the ratio of the peak amplitude and the noise height (peak-to-peak) divided by 2.5 (*σ*_2_) 68. Both SNR estimations are shown for all *in vivo* ^19^F MR measurements in healthy and EAE mice (**Table S1**). SNR_1_ was used for all data analysis. SNR_2_ is only reported in Table S1.

The time domain (TD) signal intensity was measured by calculating the y-axis intercept of the magnitude free-induction decay using a 4-th degree polynomial fit (FID fit) in MATLAB. The frequency domain (FD) signal intensity was determined by computing the integral of the MRS peak at -61 ppm (peak area). For this, we used a Lorentzian fit of the real spectrum 69 including a baseline offset and a secondary peak if the amplitude of the secondary peak exceeded SNR_1_ = 2. Only the area of the main peak was attributed to TF.

T_1_ and T_2_ were determined by mono exponential fitting of data points obtained from ^19^F MRS (T_1_) and CPMG (T_2_, three parameter fit).

### Statistical analysis

MR-data and mass spectrometry data were pooled from all experiments. ^19^F signal intensities from ^19^F MR and TF concentrations in serum, perfused brain tissue and CSF samples from HPLC/MS experiments were log transformed, and the log-normal distribution confirmed using the Shapiro-Wilk normality test. EAE disease scores were presented as mean and standard error of the mean, maximum scores as median and interquartile range and were analyzed with the Mann-Whitney U test. Bodyweight was analyzed using the t-test; the logrank test was used to analyze the time-to-onset of clinical signs. ^19^F MR signal detection over time and mass spectrometry data was analyzed using ANOVA or 2-factor ANOVA, with the Tukey post-hoc test for multiple comparisons, or with an unpaired t-test. Levene's test was used for testing for homogeneity of variance. Correlation was assessed using the Pearson correlation (R) or the non-parametric Spearman rank-order correlation (ρ), as appropriate. *p*-values < 5 % were considered significant (depicted as *p < 0.05; **p < 0.01; ***p < 0.001). Statistical analysis was performed using the statistical computing environment R (version 3.6.1, R Foundation; https://R-project.org).

## Results

### Strain differences in response to teriflunomide treatment in EAE mice

**Figure 1:**
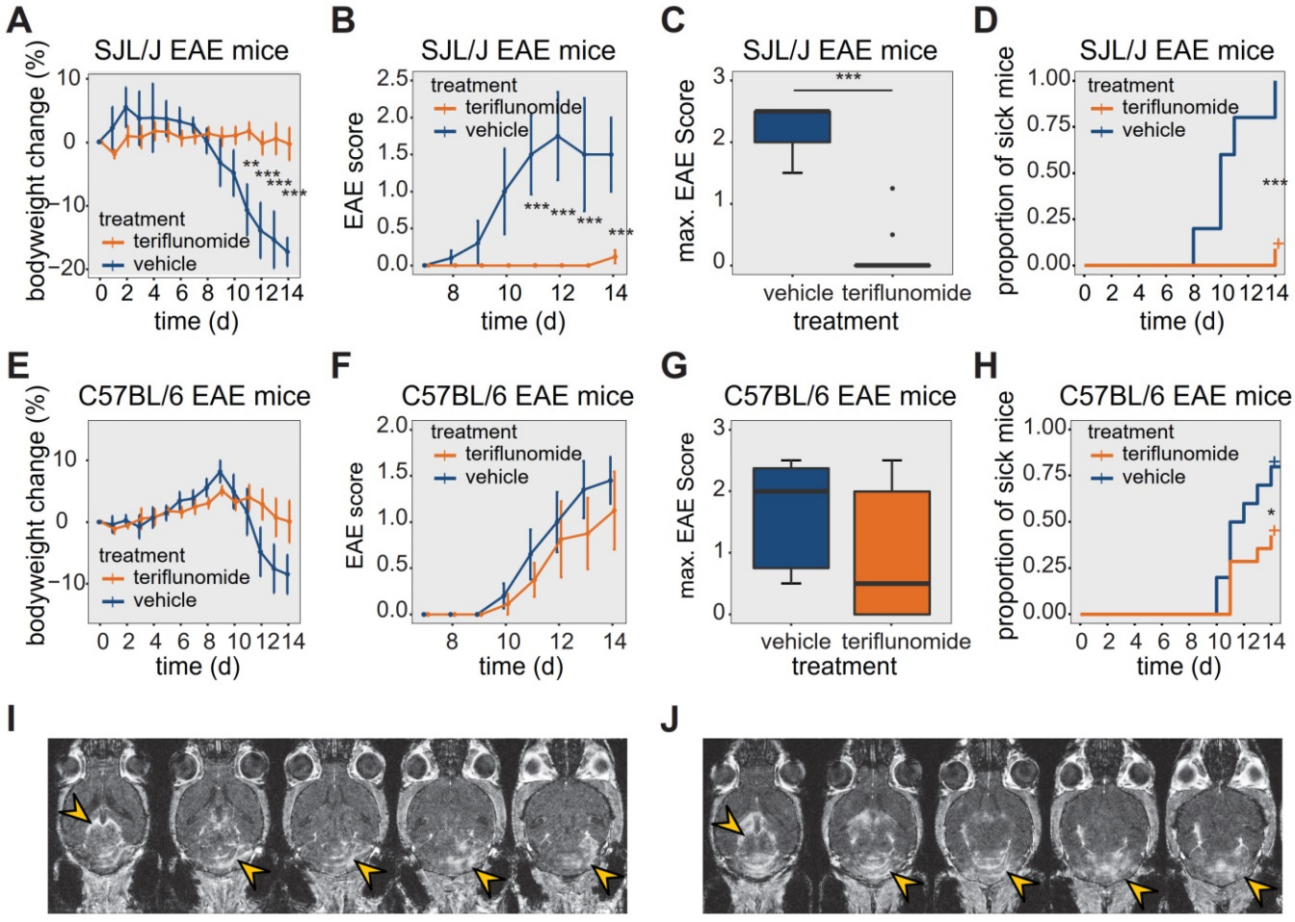
* Effect of teriflunomide treatment on the disease course of EAE in SJL/J and C57BL/6 mice*. **(A)** Change in bodyweight (Mean ± SE) over time in untreated (carboxymethylcellulose vehicle, n = 5) and TF treated (n = 12) SJL/J EAE mice. Changes in percent compared to the initial bodyweight are shown. **(B)** Mean EAE score ± SE of untreated (n = 5) and TF treated (n = 12) SJL/J EAE animals. The time axis is restricted to days with non-zero EAE score. **(C)** Maximum EAE score reached during the EAE disease course in untreated (n = 5) and TF treated (n = 12) SJL/J EAE animals. **(D)** Kaplan-Meier plot of untreated (n = 5) and TF treated (n = 12) SJL/J EAE animals depicting the time to disease onset (score=0.5) and the proportion of animals with clinical symptoms. **(E)** Change in bodyweight (Mean ± SE) over time in untreated (carboxymethylcellulose, n = 6) and TF treated (n = 9) C57BL/6 EAE mice. Changes in percent of the initial bodyweight is shown. **(F)** Mean EAE score ± SE of untreated (n = 6) and TF treated (n = 9) C57BL/6 EAE animals. The time axis is restricted to days with non-zero EAE score. **(G)** Maximum EAE score reached during the EAE disease course in untreated (n = 6) and treated (n = 9) C57BL/6 EAE animals. **(H)** Kaplan-Meier plot of untreated (n = 6) and treated (n = 9) C57BL/6 EAE animals depicting the time to onset (score = 0.5) of the EAE disease and the proportion of animals with clinical symptoms. **(I-J)** MR Images showing mild (I) and severe (J) blood brain barrier disruption using contrast agent (i.v.) and MDEFT on day 14 in two TF treated EAE mice. Lesions are indicated (yellow arrows) in the cerebellum and also in periventricular regions. Differences in body weight were analysed using Student's t-test; EAE scores and max scores were analysed using the Mann-Whitney U test; time to disease onset was analysed using the logrank test.

We studied TF treatment response in both SJL/J (**Figure 1A-D**) and C57BL/6 (**Figure 1E-H**) EAE mice. In SJL/J mice, TF treatment prevented weight loss during the EAE disease course (**Figure 1A**, n = 12, pooled from 4 EAE experiments). Control EAE mice (n = 5, pooled from 4 EAE experiments) treated with vehicle showed a substantial weight loss from day 11 post-immunization (p.i.) onward (*p* = 0.002) (**Figure 1A**). TF treatment resulted in an almost complete absence of clinical signs in SJL/J mice. TF-treated EAE mice had lower clinical scores compared to vehicle-treated EAE mice, which showed a typical disease course for SJL/J mice, reaching peak clinical score at day 12 p.i. (**Figure 1B**). In the TF-treated group, only 8% of animals showed clinical signs by day 14 p.i. compared to untreated mice (100% incidence). The maximum disease score was also different between treated (0 ± 0, median ± interquartile range, IQR) and untreated (2.5 ± 0.5, median ± IQR) EAE mice (*p* < 0.001) (**Figure 1C**). TF delayed disease onset (*p* < 0.001), which is defined as time to reach a minimum clinical score of 0.5 (**Figure 1D**).

The response of C57BL/6 EAE mice (n = 9, pooled from 3 EAE experiments) to TF treatment was less pronounced. TF-treated C57BL/6 EAE mice showed a less marked reduction in weight over time, but there was no significant difference in weight loss compared to vehicle-treated C57BL/6 EAE controls (n = 6, pooled from 3 EAE experiments) (*p* > 0.1) (**Figure 1E**). TF-treated C57BL/6 mice began to show clinical signs by day 10 p.i., and while EAE scores were generally lower than those of vehicle treated controls, there were no differences in EAE scores between the two groups (*p* > 0.5) (**Figure 1F**). Also, there was no significant decrease in maximum EAE score in TF-treated C57BL/6 EAE mice (0.5 ± 2, median ± IQR) compared to vehicle-treated (2 ± 1.625, median ± IQR) C57BL/6 EAE mice (*p* > 0.05) (**Figure 1G**). TF treatment reduced disease incidence by 65% and also delayed onset, compared to untreated mice (*p* = 0.04) (**Figure 1H**).

The extent of CNS inflammation was examined on day 14 by measuring BBB disruption using contrast-enhanced MRI (**Figure 1I-J**). We observed contrast-enhancing lesions in SJL/J EAE mice treated with TF, even in the absence of clinical signs. The extent of these lesions varied among animals, with some showing comparatively mild (**Figure 1I**) and others comparatively severe (**Figure 1J**) disruption. Contrast-enhancing lesions were particularly prominent in the cerebellum (**Figure 1I-J**) and were also present in periventricular regions (**Figure 1J**).

### Environmental factors alter the magnetic resonance properties of teriflunomide

**Figure 2:**
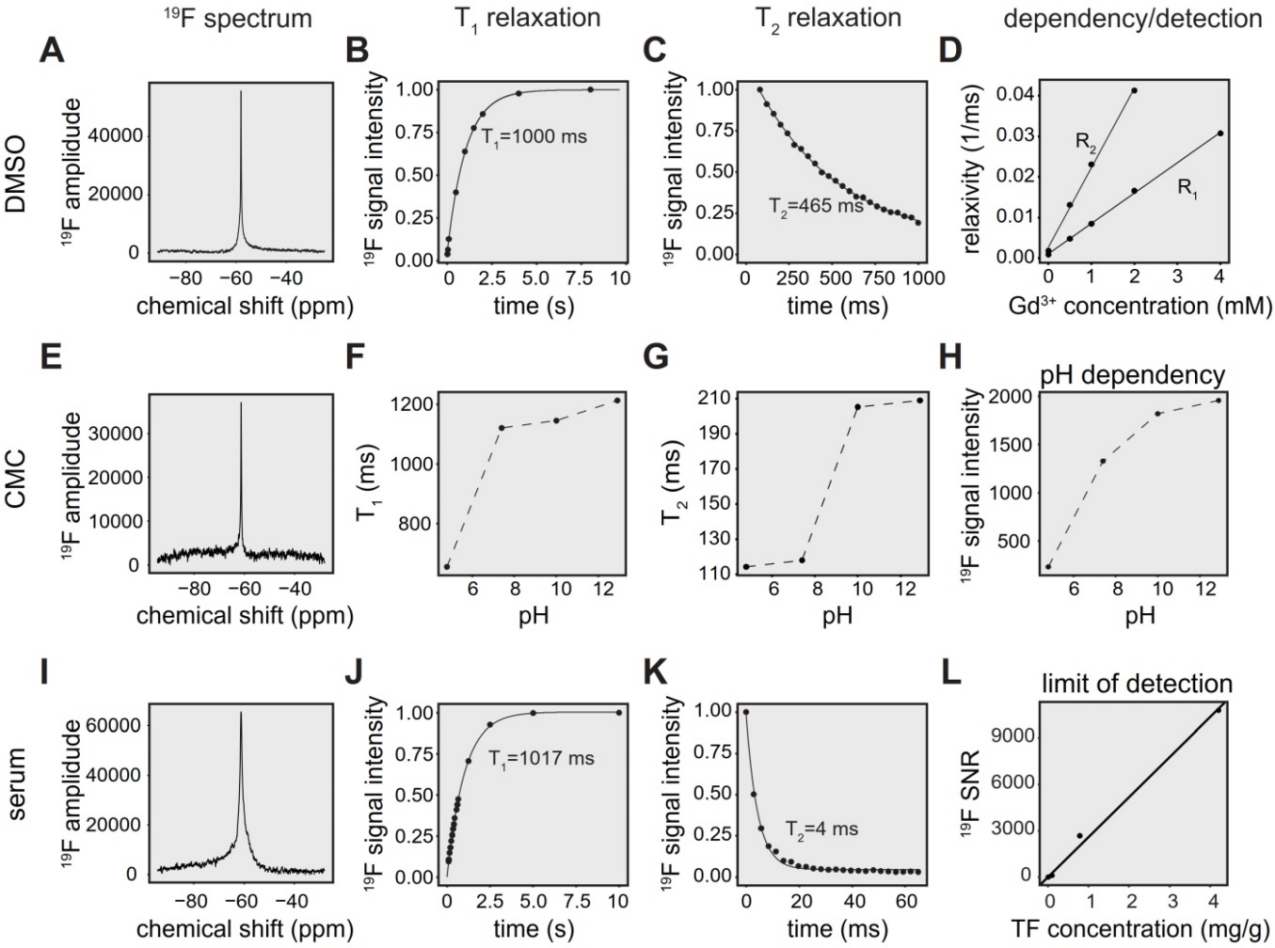
*^19^F MR characterization of teriflunomide in different chemical environments.*** (A)** ^19^F MR spectrum of TF in a DMSO phantom (global single pulse, TR=1000 ms, acquisition time = 16 s, concentration: 27.02 mg/mL in 1 mL).** (B)** Spectroscopic determination of T_1_ of teriflunomide in DMSO (T_1_ = 1000 ms). **(C)** Spectroscopic determination of T_2_ of teriflunomide in DMSO (T_2_ = 465 ms) using a CPMG sequence.** (D)** Correlation of the relaxation rates R_1_ and R_2_ (inverse T_1_ and inverse T_2_) with the concentration of the contrast agent gadopentetate dimeglumine (0.5, 1, 2, 4 mM) in DMSO (R = 0.998, p = 9.17e-6 for R_1_ and R = 0.999, p = 0.001 for R_2_). TF concentration = 27.02 mg/mL.** (E)** ^19^F MR spectrum of TF in a carboxymethylcellulose (CMC) phantom (global single pulse, TR=1000 ms, acquisition time = 16 s, concentration: 2.70 mg/mL in 1 mL). **(F)** Change of the ^19^F T_1_ with pH in CMC. **(G)** Change of the ^19^F T_2_ with pH in CMC. **(H)** Change of the ^19^F signal intensity with pH in CMC; concentration in CMC: 2.70 mg/mL in 1 mL, pH was controlled by adding HCl and NaOH.** (I)** ^19^F MR spectrum of teriflunomide in a serum phantom (acquisition time = 8 s, concentration: 1.3 mM in 1 mL).** (J)** Spectroscopic determination of T_1_ of teriflunomide in human serum (T_1_ = 1017 ms). **(K)** Spectroscopic determination of T_2_ of teriflunomide in human serum (T_2_ = 4 ms) using a CPMG sequence. **(L)** Assessment of the spectroscopic limit of detection (SNR_1_) using different TF concentrations in serum (^19^F MRS measured with a ^19^F CRP, global single pulse, TR = 1000 ms, acquisition time = 17 min).

The physicochemical properties of TF in DMSO, including chemical shift, ^19^F T_1_ and ^19^F T_2_ relaxation times are shown in **Figure 2A-C**. Changes in T_1_ and T_2_ occur with different concentrations of gadopentetate dimeglumine (**Figure 2D**). We observed a linear correlation between the inverse T_1_ (R_1_) (T_1_-relaxation times 1099 ms, 209 ms, 117 ms, 60 ms, 33 ms; Pearson R = 0.999, *p* < 0.001) and inverse of T_2_ (R_2_; T_2_-relaxation times 547 ms, 76 ms, 43 ms, 24 ms; Pearson R = 0.999, *p* = 0.001) with increasing Gd-DTPA concentrations (**Figure 2D**).

TF in CMC exhibits a single narrow peak spectrum (FWHM = 116 Hz) at ‑61 ppm (**Figure 2E**), in comparison to a peak at ‑58 ppm for experiments performed in DMSO (FWHM = 117 Hz) 70.

Both T_1_ (**Figure 2F**) and T_2_ (**Figure 2G**) were increased with increasing pH. We also observed increased signal intensity at higher pH using global single pulse spectroscopy with full relaxation (**Figure 2H**).

Compared to spectra in DMSO (FWHM = 117 Hz) 70, we observed a broader peak for TF in serum (FWHM = 528 Hz) at -61 ppm (**Figure 2I**). In addition, we characterized T_1_ (**Figure 2J**) and T_2_ (**Figure 2K**) in human serum in order to optimize pulse sequences for subsequent *in vivo* measurements. T_1_ of TF in serum was 1017 ms (**Figure 2J,** R² = 0.999), which was comparable to the T_1_ of 1000 ms in DMSO (**Figure 2B**). Conversely, T_2_ was markedly shortened to 4 ms (**Figure 2K,** R² = 0.963) in the presence of serum, which is 93-fold lower than the T_2_ of 465 ms in DMSO 70.

We obtained a detection limit for the ^19^F CRP using ^19^F MRS, validating the concentrations with mass spectrometry. At an SNR threshold of 5, the LOD was 1.9 µg/g, which is equal to 5.04e + 15 ^19^F atoms in a volume of 400 µL (**Figure 2L**).

### *In vivo* detection of teriflunomide in the abdominal region of healthy animals

Similar to TF spectra in CMC phantoms, we observed a TF peak at ‑61 ppm in healthy Dark Agouti rats (n=2) using ^19^F MRS (**Figure S1A**). Qualitatively, we discerned an initial increase in ^19^F MR signal followed by gradual decrease during the observation period of 30 minutes. We also detected TF in the abdominal region of healthy C57BL/6 mice (**Figure S1B**) 24 hours after the last drug administration, using the ^19^F CRP.

### *In vivo* detection of teriflunomide in the head region

**Figure 3:**
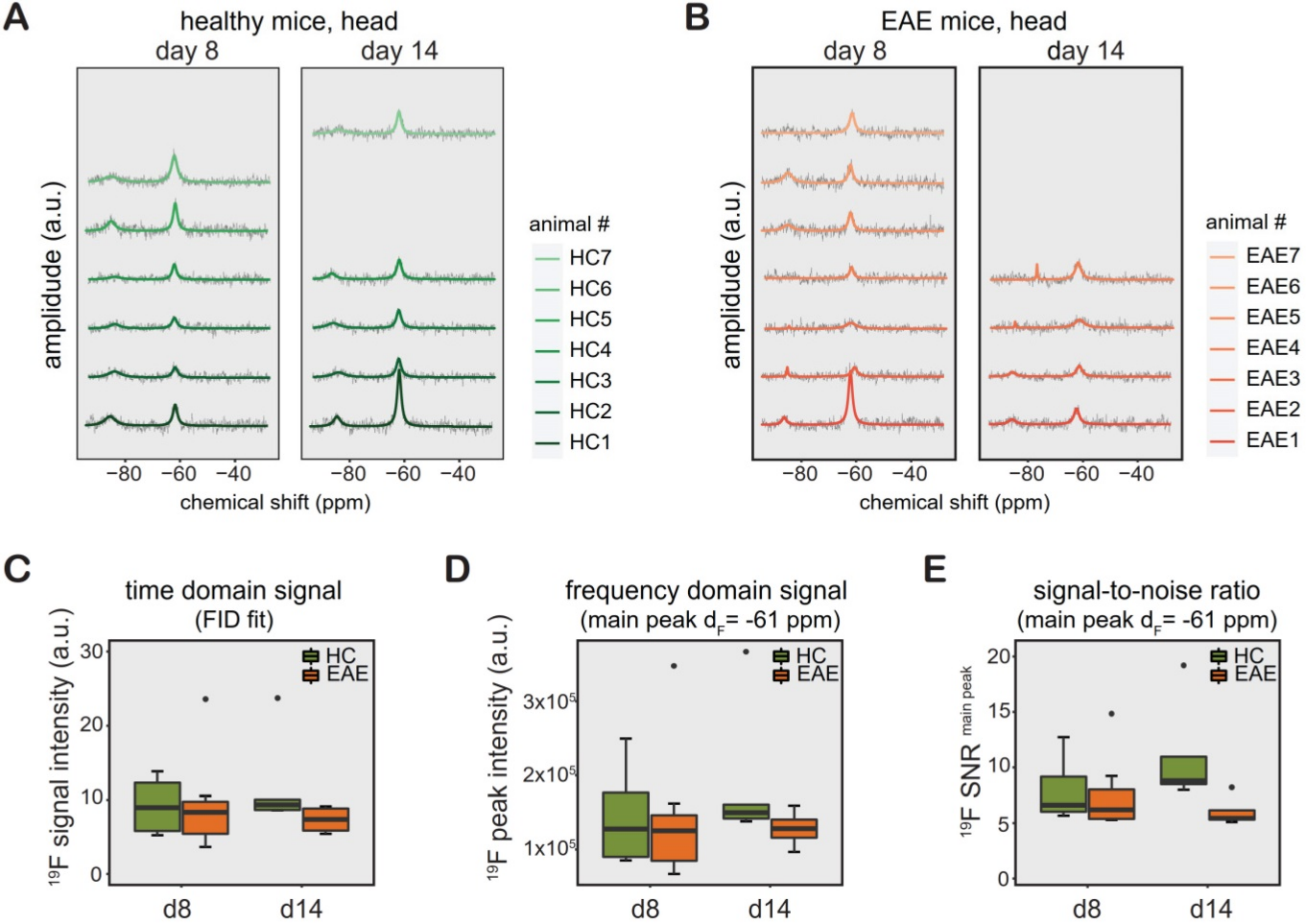
*^19^F MR detection of teriflunomide (TF) in vivo.* (**A-B**) ^19^F MR teriflunomide signal from the heads of healthy (A) and EAE (B) mice on day 8 and day 14 following the start of daily teriflunomide treatments. Measurements were performed 16-24 h after the last gavage (acquisition time = 17 min).** (C-D)** ^19^F MR signal calculated in the time domain as signal intensity from the FID fit (C) and frequency domain as peak area, integral of the main peak (δ_F_ = -61ppm) using the Lorentzian fit (D) and SNR (signal per one SD of the noise) of the same peak (E) plotted as arbitrary units for all EAE and healthy SJL/J animals for days 8 and 14.

Similar to CMC phantoms and *in vivo* measurements in the rat abdomen, we observed a TF peak at ‑61 ppm in the mouse head region. We studied changes in TF levels in TF-treated healthy mice (**Figure 3A**, n = 6 on day8, n = 5 on day14, from 1 EAE experiment), and TF-treated EAE mice (**Figure 3B**, n = 7 on day8, n = 4 on day14, from 1 EAE experiment). Differences in animal numbers between the time points were either due to technical problems (4 cases) or due to animal welfare (mice needed to be euthanized due to disease severity, 2 cases). We observed a distinct second ^19^F peak in healthy and EAE animals (-75 to -85 ppm). The -75 ppm peak was seen at a later stage of EAE and was also observed in the abdomen of EAE mice (data not shown).

The ^19^F MR signal from the processed spectra of EAE and healthy mice is represented in both TD as FID fit (**Figure 3C**) and FD as peak area (integral of the main peak) at ‑61 ppm (**Figure 3D**) and SNR_1_ of this peak (**Figure 3E**). Data from these mice are shown separately in **Table S1** (the raw data is also available as supplemental material). We did not observe any significant differences between the groups between day 8 and day 14, between EAE and healthy control mice (all *p* > 0.1) or in the pairwise comparisons (all *p* > 0.1) for all data, irrelevant whether FID fit, integral or SNR of main peak. In addition, we did not observe differences in variance of the ^19^F signal intensities (FID fit, integral or SNR) among the animals groups on any day or any of the pairwise comparisons (all *p* > 0.1).

### *Ex vivo* determination of teriflunomide levels in healthy and EAE animals

**Figure 4:**
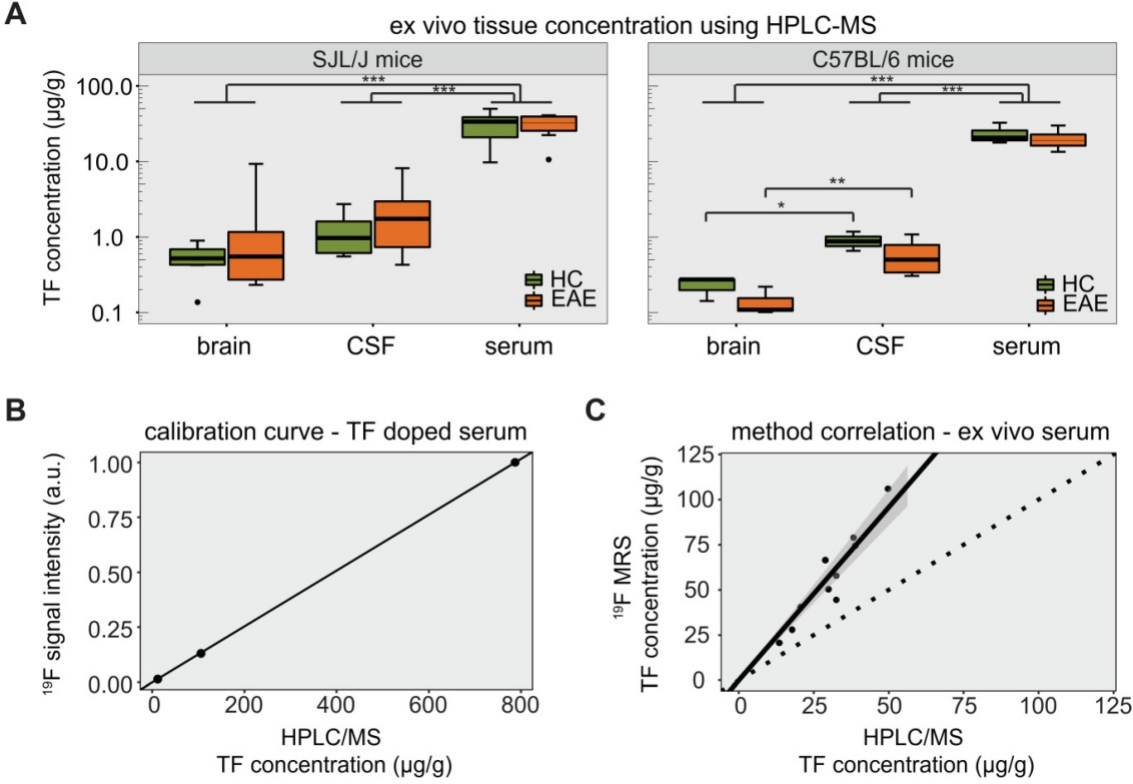
* Detection of teriflunomide by mass spectrometry.*** (A)** Plot showing the detection of teriflunomide by mass spectrometry in the brain, CSF and serum of EAE and healthy animals of both strains SJL/J and C57BL/6 on day 14 (EAE SJL/J n = 7, healthy SJL/J n = 6, EAE C57BL/6 n = 4, healthy C57BL/6 n = 3). **(B)** Calibration curve for ^19^F MRS quantification using HPLC/MS concentrations of TF dissolved in serum and the corresponding ^19^F MRS signal intensities (SNR_1_; ^19^F MRS measured with a ^19^F CRP, global single pulse, TR = 1000 ms, acquisition time = 17 min, linear fit with Spearman ρ = 1.000, p = 0.333). **(C)** Correlation of ^19^F MR signal quantification *ex vivo* in the serum of TF treated mice with concentrations measured by mass spectrometry in serum (^19^F MRS measured with a ^19^F CRP, global single pulse, TR = 1000 ms, acquisition time=1 min, Spearman ρ = 0.903, p = 0.001, dotted line with slope = 1).

The TF-derived signal was also measured in the serum of SJL/J EAE mice by ^19^F MRS (**Figure S1C**). TF concentrations in serum, CSF and perfused brain tissue were quantified by HPLC/MS for both SJL/J and C57BL/6 mice (**Table 1**). We calculated the LOD of the HPLC method to be 4.9 pg/g. In SJL/J mice there was a strong difference between biological samples (main effect *p* < 0.001), but no significant difference between healthy and EAE animals. TF concentrations in serum were an order of magnitude higher than those in perfused brain tissue or CSF, for both healthy and EAE animals (all *p* values < 0.001) (**Figure 4A, left panel**). In C57BL/6 mice, there was again a strong difference between biological samples (main effect *p* < 0.001), with TF concentrations in serum greater than those in perfused brain tissue or CSF (*p* < 0.001). Upon post-hoc comparisons, we observed that TF concentrations in the CSF were greater than in the perfused brain tissue, both for C57BL/6 EAE mice (*p* = 0.0025) and for C57BL/6 healthy control mice (*p* = 0.032) (**Figure 4A, right panel**).

We observed significant differences in the variance of TF concentrations. In SJL/J mice, these differences were seen among perfused brain, CSF and serum samples (*p* < 0.001) but not between EAE and healthy control groups when considering all *ex vivo* samples (*p* = 0.945). When comparing EAE vs. healthy controls for each tissue separately (pairwise comparisons), the variance in TF concentration was significantly greater in the EAE group in the case of brain tissue (*p* = 0.048), but not CSF and serum samples (**Figure 4A, left panel**). In C57BL/6 mice, differences in variance were also seen among perfused brain, CSF and serum samples (*p* < 0.001). There was again no difference in TF variance between EAE and healthy controls (*p* = 0.406), even when performing the pairwise comparisons for each tissue (**Figure 4A, right panel**).

The HPLC/MS quantification of TF concentrations was important to establish the ground truth for validating *in vivo* ^19^F MRS data. A calibration of FD ^19^F MRS signal intensities with HPLC/MS concentration values in mouse serum (spiked with different TF dilution) is shown in **Figure 4B**. The resulting linear fit (Spearman ρ = 1.000, *p* = 0.333), was used to estimate the concentration from the FD ^19^F MR signal intensity. Compared to the HPLC/MS quantification, TF concentrations estimated from ^19^F MRS were elevated with a maximum relative deviation of 130% and a mean relative deviation of 83% (**Figure 4C**). However, TF concentrations determined by ^19^F MRS showed a clear correlation with concentrations determined by HPLC/MS (Spearman ρ = 0.903, *p* = 0.001).

## Discussion

In this study, we show that non-invasive^ 19^F MR methods can be used to detect TF *in vivo*. *Ex vivo* HPLC/MS analyses confirmed the availability of TF in the CNS at pharmacologically relevant concentrations 56. The therapeutic effect was strain-dependent, being less pronounced in C57BL/6 mice. This could be attributed to the diverging pathology that both strains present during the course of an EAE: in SJL/J mice the pathology is mainly localized to the brain, in C57BL/6 mice lesions are mostly prevalent in the spinal cord 71. EAE in SJL/J mice presents as a relapsing-remitting disease (similar to RRMS patients). EAE in C57BL/6 mice follows a chronic disease progression without remissions and relapses (similar to progressive/secondary progressive MS). These strain differences were behind the rationale for studying different mechanism of action of DMDs for the treatment of RRMS and P/SPMS 56. Different responses to TF treatment among species and strains could be also attributed to differences in target binding potencies 72 or immune cell susceptibilities 56.

Despite increasing concerns regarding the risks of long-term deposition in the brain, contrast-enhanced MRI remains a key tool for diagnosis and differential diagnosis 73-75. In this study we observed contrast-enhanced brain lesions at the expected time of peak disease, even in asymptomatic TF-treated EAE mice. This underscores the critical role of MRI for early detection of pathology in MS and EAE, even prior to the occurrence of clinical signs and that clinical scoring alone is not sufficient to fully assess the disease status 58.

In addition to peripheral effects and mechanisms of action, many DMDs for MS are expected to work within the CNS. Thus a non-invasive method that studies drug distribution in the CNS would be a useful tool in MS drug development and in treatment monitoring. Hence, we are addressing an area of major interest in MS that could benefit from new studies investigating therapies and their distribution *in vivo*. Nevertheless, the *in vivo* detection of ^19^F compounds with ^19^F MR methods remains challenging. This is primarily due to the low drug concentrations available in the human body 70. Additionally, technological challenges in terms of hardware sensitivity and measurement precision and accuracy limit swift transitions to clinical applications.

^19^F MR spectroscopy techniques have been used for several years to detect fluorinated drugs in small animals 33-38 and humans 39-45. The chemotherapeutic agent 5-Fluorouracil (5-FU) has been studied by ^19^F MRS in tumor-bearing rats 34 and patients with head and neck tumors 45 and more recently it was detected by ^19^F MRI in tumor bearing mice using high drug doses and fast spin echo sequences 38. ^19^F MRS imaging (MRSI) of fluvoxamine and fluoxetine was performed in patients with major depressive disorder who were on long term treatment with these drugs 42. Alternatively, therapeutic compounds containing cytosine (e.g. the neuroprotective drug citicoline or the anticancer drug gemcitabine) can be detected via chemical exchange saturation transfer (CEST) MRI; these molecules contain exchangeable protons that can be selectively saturated and then detected indirectly through the water signal. Recently, the potential of CEST MRI to detect these therapeutic compounds *in vivo* has been shown 76, 77.

Compared to studies investigating fluorinated drugs at high doses 38, 44, in our present study we administered therapeutic doses of the fluorinated drug, which previously had shown an influence on the disease course in SJL/J EAE mice 56. Additionally, we did not only acquire ^19^F MR signals right after administration of the drug as has previously been done 36, 78, 79, but we also detected accumulated TF levels over time, and during pathology.

Furthermore, in this study we characterized the MR properties of TF in serum, DMSO and CMC and at varying pH, to assess alterations in the physicochemical and MR properties, which are important to consider during interpretation of data. The pH in different compartments has a known impact on the solubility, binding kinetics and hence bioavailability of drug molecules 80. pH variations result in different protonation of molecules, changing their MR properties, such as chemical shift 81 and relaxation times 82-86. pH changes can also effect drug solubility, e.g. at low pH only a fraction of TF is dissolved and thus detected by ^19^F MR, the precipitated portion will not contribute to the MR signal. Furthermore, pH could also affect the properties of the CMC support matrix. The stomach environment could alter its protonation state and therefore its solubility, thereby affecting the ^19^F MR properties of TF 87, 88.

While the T_1_ of TF in serum was comparable to that in DMSO, the T_2_ was substantially shortened in serum, which is also indicated by a broader TF peak 89 in serum, when compared to DMSO. Similar to micelles and nanoparticles 90, CMC could perhaps bind to serum proteins, although one would assume that the drug will be mostly bound to serum proteins in the blood stream. In any case, a shortening of T_2_ if caused by drug serum binding (or CMC serum binding) makes signal detection more challenging when using standard pulse sequences*.*

Here, we used a three-parameter exponential fit, taking an offset of the signal decay into account until reaching the level of noise. Potentially, another fraction of TF exhibiting a different relaxation behavior might contribute to the acquired signal, which however, cannot be distinguished in the experiments performed here. Conversely, an increase in TF MR signal can be expected in short TR measurements in the presence of gadolinium-based contrast agents due to a reduction in T_1_ saturation effects. This has implications for neuroinflammation since TF is likely to localize at sites of inflammatory activity in the brain or the spinal cord, and thus its proximity to gadolinium-enhanced lesions in the CNS might increase its detection.

We previously showed that temperature can influence the MR parameters of TF 70. Here, ^19^F MR signals increased with increasing pH. Differences in spectral widths between TF in DMSO, CMC and serum can be explained by environmental effects such as different solubility of the drug in the medium and protein-binding effects (99% of TF is plasma protein bound) 91.

As a first step towards studying drug distribution *in vivo*, we measured the ^19^F MR signal in the abdominal region of healthy rats. The ^19^F MR signal was acquired directly after TF administration into the stomach; we assume that most of this signal originates from the stomach during the first few minutes. Changes in ^19^F signal in rats following oral administration can be attributed to pH and temperature changes in the stomach that may alter the solubility or binding of TF to CMC. We hypothesize that pH might play an ambivalent role in different environments e.g. in the stomach, in CMC or in DMSO with respect to solubility and consequently TF detection. The decreasing trend in ^19^F signal after the six-minute measurement could suggest a gradual influence of the acidic pH in the stomach on signal intensity but also a gradual distribution to the intestinal compartment and absorption into the blood circulation. We expect that the ^19^F MR signal from the abdominal region in EAE mice, measured 24 h after the last drug administration originates primarily from the liver (highest concentration of TF after blood) 92.

When studying the *in vivo* ^19^F MR signal of TF in the head region, we observed no changes between day 8 and day 14 p.i. In patients, TF has a half-life of approximately 15 days 51, 93 administered at a dose of 14 mg per day (circa 250 µg/kg). This corresponds to a mouse dose of circa 3 mg/kg 60. In the current study and previous ones 55, 56 mice received 3-10 times this dose, which is needed to have an effect on the disease course 56. Leflunomide, the prodrug of TF, reaches steady state in 7 weeks when administered orally at a daily dose of 20 mg (circa 350 µg/kg). If linear pharmacokinetics are assumed 94, the steady state of TF dose used in mice is expected to be reached earlier than day 8.

Interestingly, we observed a second peak in the range of -75 to ‑85 ppm alongside the main TF peak at ‑61 ppm in healthy and EAE mice, and in their *ex vivo* sera, but not in *in vivo* experiments carried out soon after drug administration in the rat. We assume that this peak is a TF metabolite. While we are not aware of any specific TF metabolites that resonate at this range, we are certain that this is not a contamination since it was not reproduced in our phantom experiments and our animals had not been exposed to any other ^19^F compounds. We believe this second peak surely warrants further investigation and might be valuable to further pharmacological research.

The major metabolite of TF in human plasma is 4-trifluoro-methylaniline oxanilic acid (4-TMOA) 51, 95. This metabolite has a chemical shift of -59.7 ppm and would overlap with the TF parent compound 96. Other metabolites such as mono-oxidated TF sulfate, 4-trifluoromethylaniline 2-hydroxy-maionanlc malonamic acid and its sulfate were identified in urine, and mono-oxidated TF sulfate and mono-oxidated TF in feces 95.

The chemical shifts of most CF3 groups lie within the range of -60 to -80 ppm. A more negative chemical shift would indicate increased shielding of the CF3 group, which can occur as a result of branching near the CF3 group or close proximity to hydrogen bond donors 97. In human subjects the total amount of these metabolites in plasma is lower than 1 % of the parent compound (in contrast to urine and feces) and probably not detectable *in vivo* 95, 98. However, this might also be different in mice. Different metabolic rates and processes can be an explanation of this finding. Even the EAE pathology might have an impact due to changes in metabolic processes during inflammation 99 and warrants further investigation in future studies.

In a rat EAE model, TF distribution to the brain was shown by whole-body autoradiography 92, whereas no TF could be detected in the brains of EAE mice when using MALDI-MS 100. The *in vivo* ^19^F MR signal that we acquired in the head region of healthy and EAE mice with ^19^F MRS could reflect TF signals in the blood, CSF, brain parenchyma, or perhaps even infiltrating immune cells that are causing the pathology. Since we did not observe any significant differences in TF signal between the healthy controls and EAE mice *in vivo*, there is no evidence of pathology-related alterations in the drug distribution into the head region.

The results from mass spectrometry measurements also did not show significant differences in TF concentrations in perfused brain tissue, CSF and serum between EAE mice and healthy controls. Nevertheless, TF was detectable in CSF and perfused brain tissue in both SJL/J and C57BL/6 mice, though these were significantly lower than serum concentrations. This distribution pattern could reflect the route of the drug from the systemic circulation to the brain via the CSF or the vasculature.

The observed interindividual differences in TF levels are consistent with a TF study performed in patients where steady state plasma concentrations were in the range 7.6-14.8 mg/L and 11-16.9 mg/L following at least 8 weeks daily intake 93.

One caveat of the study was that we could not perform automatic power adjustments during the *in vivo* measurements in the mouse head, due to low SNR; instead we performed short measurements with different reference power settings on the living animal to determine the optimal reference power to reach the 90 ° flip angle. Nonetheless the uncertainty in the flip angle due to potential differences in the positioning of the animal or coil filling factors could be a potential source of variability in the ^19^F detection. We did not use a reference tube during the *in vivo* measurements for several technical (overlapping signals, potential signal losses), physical (complex *in vivo* setup and limited space) and animal welfare related (breathing obstruction) reasons.

A comparison between the ^19^F MRS method and the HPLC/MS method in *ex vivo* serum samples showed a linear correlation. However, there were deviations between HPLC/MS and ^19^F MRS concentration estimations; one could attribute these either to an overestimation by the ^19^F MRS method or an underestimation by the HPLC/MS method. The proportion of metabolite to parent compound might be higher in mice than in human subjects. Therefore, one could speculate, that the ^19^F MRS peak at ‑61 ppm overlaps with a significant amount of its metabolite. Potentially, metabolites detected by ^19^F MRS are not quantified by the HPLC/MS method and thereby could explain the deviation in the concentration estimation in both methods. Alternatively, differences in the properties of TF between spiked and *ex vivo* serum samples (e.g. differences in protein composition, conductive properties) might be a source for this deviation. Still, both methods were shown to correlate with each other and indicate that TF concentrations could be measured with ^19^F MRS in future studies, possibly even as non-invasive tool *in vivo*.

While there are still limitations in terms of technological development — in particular with regard to exact calibration of reference power due to low ^19^F amounts — we highlight here the usefulness and general feasibility of this approach for studying the biodistribution of fluorinated drugs. In this study, we needed to address several challenges for detecting TF *in vivo*. The quadrature, cryogenically cooled surface coil that we used in this study confers a theoretical increase in sensitivity of 40% 101 compared to a linear coil, but prohibits a dual-tunable feature that accommodates ^1^H imaging.

To distinguish the distribution of TF in different brain regions ^19^F MR imaging or localized ^19^F MR spectroscopy would be highly valuable. This is possible when studying neuroinflammation with ^19^F MRI and perfluoro-15-crown-5-ether nanoparticles; the ^19^F MR signal in the CNS and associated lymphatic system is sufficient for single-voxel spectroscopy e.g. PRESS (Point RESolved Spectroscopy) 61 and ^19^F MR imaging, even when using a RT coil 61. This is not the case for small molecules such as TF that are available in much smaller quantities in the CNS. Understanding the specific ^19^F MR properties of the drug of interest will allow the choice and tailoring of appropriate MR pulse sequences. While determining the specific origin of signals detected with non-localized MR spectroscopy is not possible, hypotheses on the origin of signals could be verified by using single voxel spectroscopy. Nevertheless, due to a low T_2_ of TF in serum as well as the low TF concentrations expected *in vivo* at a therapeutic level, localized single-voxel MR spectroscopy or MRSI are not trivial techniques to be applied.

In this study we characterized the MS drug teriflunomide in phantom experiments and *in vivo* in the animal model of MS. The ^19^F CRP significantly boosts SNR compared to other available RF coil technologies and enabled the *in vivo* detection of TF-derived ^19^F MR signals in EAE mice within a short time 62. However, more technological developments are needed to further boost ^19^F MR signal sensitivity to ultimately achieve drug quantification within specific tissue compartments 102. The combination of multiple approaches such as using cryogenically cooled RF coils 62, higher magnetic fields 103 and methods to accelerate data acquisition such as compressed sensing 104 will be key to achieve this goal and allow monitoring drugs *in vivo* with ^19^F MRI.

## Figures and Tables

**Table 1:** Concentrations of teriflunomide in µg/g detected via HPLC/MS from *ex vivo* samples from EAE and healthy SJL/J and C57BL/6 mice (median ± interquartile range)

	SJL/J		C57BL/6	
	EAE (n = 7)	Healthy (n = 6)	EAE (n = 4)	Healthy (n = 3)
Serum	32.5 ± 13.8	33.8 ± 17.9	19.0 ± 6.8	20.6 ± 7.4
CSF	1.7 ± 2.2	1.0 ± 1.0	0.5 ± 0.5	0.9 ± 0.3
Brain	0.6 ± 0.9	0.5 ± 0.3	0.1 ± 0.0	0.3 ± 0.1

## Abbreviations

BBB: blood brain barrier
CFA: complete Freund's adjuvants
CMC: carboxymethylcellulose
CNS: central nervous system
CRP: cryoprobe
CSF: cerebrospinal fluid
DMD: disease modifying drug
EAE: experimental autoimmune encephalomyelitis
FWHM: full-width half maximum
HPLC/MS: high performance liquid chromatography mass spectrometry
LOD: limit of detection
MOG: myelin oligodendrocyte glycoprotein peptide
MR: magnetic resonance
MRI: magnetic resonance imaging
MRS: magnetic resonance spectroscopy
MS: Multiple Sclerosis
PLP: proteolipid protein peptide
PRESS: Point RESolved Spectroscopy
RRMS: relapse-remitting Multiple Sclerosis
SNR: signal-to-noise ratio
TF: teriflunomide
