# Supplementary material for: In vivo detection of teriflunomide-derived fluorine signal during neuroinflammation using fluorine MR spectroscopy
Source: Theranostics. 2021 Jan 1;11(6):2490–504. doi: 10.7150/thno.47130 (PMC7806491; doi:10.7150/thno.47130)
Supplement: Supplementary file 1 — Supplementary figures and tables. [file thnov11p2490s1.pdf]

Supplement

In vivo detection of teriflunomide-derived fluorine signal during neuroinflammation using fluorine MR spectroscopy

Christian Prinz, Ludger Starke, Jason M. Millward, PhD, Michael Rothe, PhD, Paula Ramos Delgado, Helmar Waiczies, PhD, Andreas Pohlmann, PhD, Ariane Fillmer, PhD, Marc Nazaré, PhD, Friedemann Paul, MD, Thoralf Niendorf, PhD, Sonia Waiczies, PhD

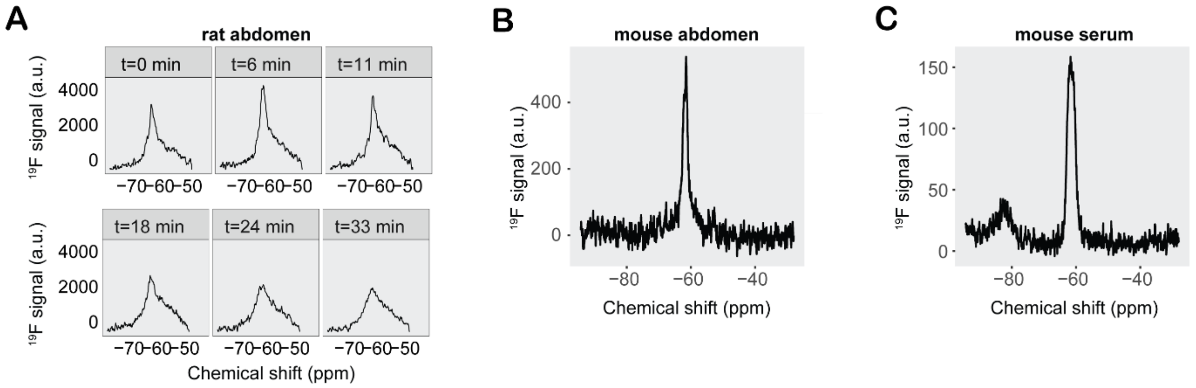

Figure S1. *^{19}F MR detection of teriflunomide (TF) in vivo and ex vivo.* **(A)** In vivo ^{19}F MR teriflunomide signal from the rat abdomen following TF administration to the stomach via gastric intubation. Global single pulse spectroscopy was used to measure the ^{19}F MR signal at 6 time points over 30 min (acquisition time=6 min 24 s). **(B)** In vivo ^{19}F MR teriflunomide signal from a healthy C57BL/6 mouse abdomen after TF treatment for 14 days (^{19}F MRS measured with a ^{19}F CRP, global single pulse, TR = 1000 ms, acquisition time = 17 min, 24 h following last TF administration). **(C)** ^{19}F MR teriflunomide signal from a SJL/J mouse ex vivo serum sample (^{19}F CRP, acquisition time = 2 h 16 min).

Table S1. *In vivo ^{19}F MR spectroscopy data.* Comprehensive data from all mice on days 8 and 14.

Animal	Day	FID fit [a]	Integral [b]	FWHM [c]	σ_1	σ_2	Amplitude [d]	SNR_1 [e]	SNR_2 [f]
EAE1	8	23.6	347853.4	671.9	20.7	51.3	307.7	14.8	6.0
EAE2	8	5.0	75917.1	844.5	16.1	37.0	86.9	5.4	2.3
EAE3	8	3.6	93509.1	1662.5	13.4	35.8	70.9	5.3	2.0
EAE4	8	5.8	67053.5	617.5	17.6	40.2	94.0	5.3	2.3
EAE5	8	8.3	130330.5	739.6	19.5	59.5	121.0	6.2	2.0
EAE6	8	9.0	125170.9	698.4	21.8	52.2	148.3	6.8	2.8
EAE7	8	10.5	162204.8	839.0	16.0	35.5	147.4	9.2	4.2
HC1	8	11.0	151852.1	743.9	19.6	42.8	127.1	6.5	3.0
HC2	8	5.5	85158.2	856.5	16.8	40.6	98.8	5.9	2.4
HC3	8	5.2	85623.1	836.0	14.0	33.6	79.6	5.7	2.4
HC4	8	6.9	103213.6	702.4	16.5	41.0	111.3	6.7	2.7
HC5	8	12.7	184937.6	690.7	20.4	45.6	203.3	10.0	4.5
HC6	8	13.8	249641.6	970.1	16.1	39.3	205.2	12.7	5.2
EAE1	14	9.1	134138.0	882.8	21.4	47.5	117.0	5.5	2.5
EAE2	14	6.0	96537.0	936.5	16.3	31.1	88.0	5.4	2.8
EAE3	14	5.4	122565.0	1591.9	15.4	40.3	78.3	5.1	1.9
EAE4	14	8.7	159295.8	969.9	15.7	34.1	128.8	8.2	3.8
HC1	14	23.7	367080.4	667.6	20.1	44.2	386.8	19.2	8.7
HC2	14	8.6	141896.2	793.5	14.2	40.5	125.3	8.8	3.1
HC3	14	8.6	138124.0	792.6	14.4	38.4	122.5	8.5	3.2
HC4	14	9.3	149673.1	801.4	17.4	33.9	139.1	8.0	4.1
HC7	14	10.0	160734.8	737.7	13.9	33.6	152.4	11.0	4.5

[a] signal intensity calculated from the y-axis intercept of the free-induction decay
[b] integral of the Lorentzian fit of the ^{19}F MRS peak at -61 ppm
[c] full width half maximum of the ^{19}F MRS peak at -61 ppm
[d] amplitude of the ^{19}F MR spectrum
[e] Signal-to-noise-ratio determined according to Kreis, et al. [1]
[f] Signal-to-noise-ratio determined according to Günther, et al. [2]

σ_1: noise level (1 standard deviation)
σ_2: noise level determined with the peak-to-peak method

References

1. Kreis R, Boer V, Choi IY, Cudalbu C, de Graaf RA, Gasparovic C, et al. Terminology and concepts for the characterization of in vivo MR spectroscopy methods and MR spectra: Background and experts' consensus recommendations. NMR in biomedicine. 2020: e4347.

2. Günther H. NMR spectroscopy : basic principles, concepts, and applications in chemistry; 2013.
